# Effects of Neuromuscular Electrical Stimulation (NMES) on salivary flow in healthy adults

**DOI:** 10.4317/jced.56572

**Published:** 2020-08-01

**Authors:** Joji Koike, Shinji Nozue, Yoshiaki Ihara, Koji Takahashi

**Affiliations:** 1 DDS, PhD. Division of Oral Rehabilitation Medicine, Department of Special Needs Dentistry, School of Dentistry, Showa University, Tokyo, Japan

## Abstract

**Background:**

Neuromuscular electrical stimulation (NMES) is a method used for enhancing suprahyoid muscle activity and is widely applied as a treatment for dysphagia. Patients often complain of saliva pooling in the pharynx during NMES. Therefore, the purpose of this study was to investigate the changes in salivary flow during NMES.

**Material and Methods:**

Twenty healthy adults participated in this study. Electrical stimulation was applied at constant strength for 60 minutes to the suprahyoid muscles using VitalStim®. Participants were examined under three conditions of NMES: sensory threshold plus 75% of the difference between sensory and pain thresholds (75% Stim), SensoryStim, and Sham. Saliva collections, using a 10-min spitting method, were performed seven times: before stimulation (S1), during stimulation (S2-S6), and 5 min after stimulation ended (S7).

**Results:**

Significant differences were observed in saliva flow between S1 and S7, as well as S2 and S7 in 75% Stim.

**Conclusions:**

This study indicates that an increase in saliva flow was promoted after NMES. Therefore, NMES may have effects on patients with xerostomia.

** Key words:**Neuromuscular electrical stimulation, suprahyoid muscle, sensory threshold, pain threshold, saliva flow.

## Introduction

Dysphagia is a swallowing disorder caused by neurological diseases including stroke, Parkinson’s disease, multiple sclerosis, dementia, motor neuron diseases, cerebral palsy, brain tumors, and myasthenia gravis. Abnormalities in swallowing function are observed in more than 28-65% of acute stroke patients (1,2) and in more than 30% of Parkinson’s disease patients (3-5). Dysphagia is also caused by congenital malformations including cleft lip and palate, and other congenital diseases with malformations and / or functional deficits. Most post-treatment head and neck cancer patients also demonstrate a degree of dysphagia.

Japan is a country with one of the longest life expectancies. There is a large population of aged persons requiring nursing care including management of dysphagia in Japan. The rehabilitation techniques for management of dysphagia are classified into direct or indirect training. Direct training is a rehabilitation technique applied to dysphagic patients who can perform oral intake under appropriate guidance for preventing aspiration or penetration. Representative guidance for preventing aspiration or penetration includes postural techniques and changing the amount or type of bolus material. Using these approaches, dysphagic patients are able to perform safe swallows without aspiration/penetration by changing the velocity and/or direction of bolus flow. Conversely, indirect training involves exercise procedures to improve neuromuscular function for acquiring safe swallows. Indirect training encompasses various exercises and strategies focusing on strengthening oral, maxillofacial, pharyngeal, laryngeal, cervical, truncal, and respiratory neuromuscular mechanisms for achieving safe swallowing.

Neuromuscular electrical stimulation (NMES) is a strategy to strengthen weak muscles by electrical stimulation. NMES activates muscular contraction and is applied in a wide range of medical fields. Recently, NMES was applied in the field of dysphagia management (6,7). NMES is used to strengthen the suprahyoid muscle complex for improving laryngeal elevation and upper esophageal sphincter (UES) opening (8). Studies have verified the effectiveness of NMES for improving dysphagic conditions in stroke and Parkinson’s disease patients (6,9-12).

In clinical settings, patients with dysphagia often complain about saliva overflow while receiving NMES treatment targeting the suprahyoid muscle complex. However, there is a lack of studies focusing on the relationship between NMES and salivary flow, and the effects of NMES on salivary flow remain unclear.

It has been reported that salivary flow is altered with circadian rhythm and degree of stress (13). Further, aging, head and neck cancer treatments, various types of medications, side effects of medical conditions (including Sjögren’s syndrome, Alzheimer’s disease, diabetes, anemia, rheumatoid arthritis, hypertension, Parkinson’s disease, stroke, and mumps) can cause reduction in salivary flow (14). Moreover, reduction in salivary flow is related to disorders of taste (14), chewing, and swallowing difficulties (dysphagia) (15). Treatments for reduced salivary flow include the adaptation of oral moisturizing agents (16), massage of major salivary glands including the parotid glands the submandibular glands (17), and changes in causative drugs (18). These treatments are adapted in patients with xerostomia. Similarly, reflective salivary flow caused by NMES affecting the submandibular glands may have an effect on patients with xerostomia. This hypothesis should be investigated to assess the effects of NMES applied to patients with xerostomia. The purpose of this study was to evaluate whether NMES to the submandibular region would directly promote salivation. We investigated changes in salivary flow under three conditions of NMES. Further, because salivary flow changes according to degree of stress, we investigated salivary cortisol levels under three conditions of NMES to examine the influence of stress.

## Material and Methods

-Subjects

Twenty healthy adult volunteers (nine males, mean age ± SD = 27.9 ± 3.8 years) participated in this study as subjects. Exclusion criteria were those with salivary gland disease, those who took medications which influenced salivation, and smokers (19). Written informed consent was obtained from each participant. This study was approved by the Showa University dentistry hospital clinical trial screening committee (DH2016-15).

-Neuromuscular electrical stimulation

**Figure 1:**
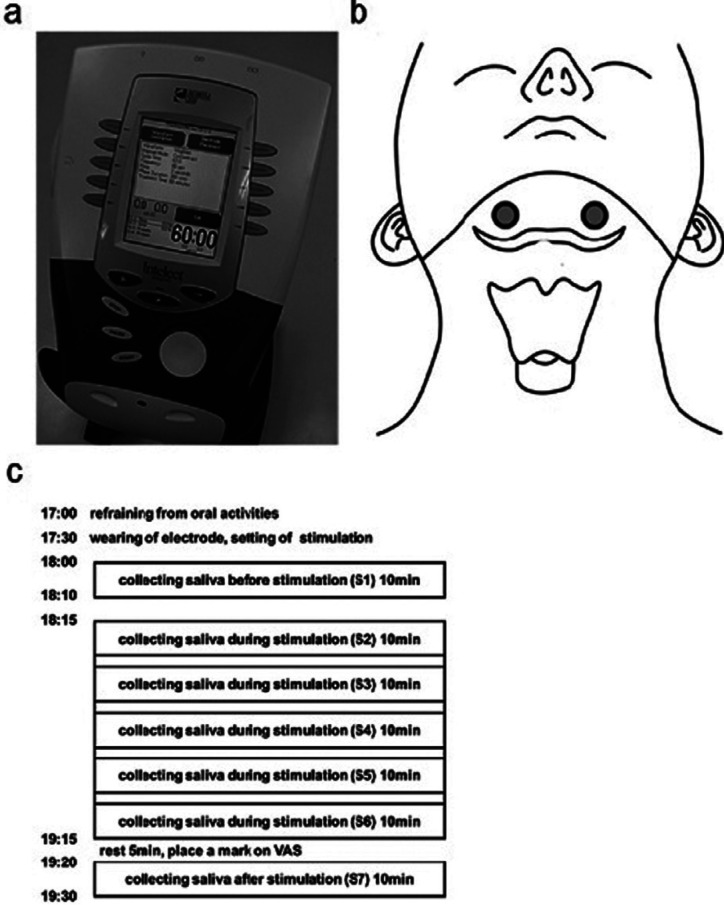
Setting of NMES. a) VitalStim® (No.59000, Chattanooga Groupe, Hixson, TN) was used as an NMES device. b) A bipolar electrode placed on the bilateral suprahyoid muscles. c) Experimental schedule. All experiments we performed according to an equivalent schedule. Saliva collections were performed seven times: before stimulation (S1), during stimulation (S2-S6), and 5 minutes after the end of stimulation (S7). There was an interval of 2 minutes 30 seconds between collection during stimulation (S2-S6).

VitalStim® (No. 59000, Chattanooga Group, Hixson, TN, USA) was used as the NMES device (Fig. 1a). VitalStim® delivered biphasic square pulse stimulation using a bipolar electrode placed on the skin over the submental region (Fig. 1b). Electrical stimulation was fixed with 80 Hz biphasic square pulse frequency and 700 μs pulse width. Each subject received three following stimulation conditions: 1) 75% Stim (20), the sensory threshold plus 75% of the difference between sensory and pain thresholds (21); 2) Sensory Stim, the sensory threshold; 3) Sham, stimulation strength 0. The strength of stimulations was determined as follows. The sensory threshold was defined as the magnitude of current at which each subject felt electrical stimulation for the first time while the electrical current increased every 0.1 mA from 0 mA. The pain threshold was defined as the magnitude of current at which each subject felt pain or felt like their skin was grabbed for the first time while the electrical current increased every 0.1 mA beyond the sensory threshold. The experiment was performed under one condition each day and was interrupted more than once a day. A total of three different conditions was performed for each subject.

-Data collection

Data including age, sex, height, weight, and body mass index (BMI) of each participant were collected. In addition, body temperature, systolic blood pressure, and diastolic blood pressure were measured before and after the stimulation. All measurements were performed by a single evaluator.

-Heart rate

Heart rate was measured using portable pulse oximeter® (Nihon Koden, Tokyo, Japan) and recorded every 10 minutes from the start of stimulation to the end of the session.

-Evaluation of discomfort and pain

Visual Analogue Scale (VAS) was used to evaluate discomfort and pain from electrical stimulation (22). After stimulation, each subject was instructed to make a mark on a VAS straight line from 0 cm to 10 cm. All subjects were informed that 0 cm meant “no discomfort or no pain” and 10 cm meant “ the strongest discomfort or the most painful they had ever experienced “.

-Experimental procedure 

All experiments were performed according to the same time schedule to avoid possible effects of homeostatic neural activities. The details of the time schedule were as follows (Fig. 1c). Brushing teeth was performed more than 1 hour before the beginning of the experiment. Eating, drinking, mouth rinsing, mouth cleaning, active conversation, and exercise were prohibited after brushing teeth (23). The measurement environment was kept under the same conditions throughout this study which included blocking the ambient noise, setting the room temperature at 24ºC, and recording the humidity of the room. Subjects were instructed to sit down on a chair and stay quiet during the experiment. The male subjects were instructed to shave their mustaches beforehand. All participants were instructed to clean the targeted skin with a special cloth (COVIDIEN® Pre-TENS Skin Prep Wipes, Mansfield, MA, USA). The electrode was attached to the targeted skin of the subjects 30 minutes prior to beginning the experiment. The electrode was placed on the skin over the suprahyoid muscle complex palpated at the middle of the inferior border of the mandible and superior border of the hyoid bone (Fig. 1b). The stimulation conditions were then set. Saliva was collected using the spitting method (24,25). Saliva was collected for a 10-minute period. Before the beginning of saliva collection, subjects were instructed to sit on the chair and swallow residual saliva in their oral cavity. Subsequently, subjects were instructed to slightly open their mouth and to drip their saliva into a plastic cup (Falcon® 50 mL) from the lower lip. In the last few seconds of the 10-minutes period, subjects were instructed to spit out residual saliva in the mouth into the plastic cup. No other voluntary movements of the oral musculature were made during the collection of the saliva. The saliva was kept in the plastic cup and measured using a measuring cylinder. Before the stimulation, saliva was collected from 18:00. After a 5-minute rest, the stimulation was started. Then, the stimulation conditions were fixed throughout all experiments. Saliva was collected after the start of stimulation, and saliva collecting procedures were repeated five times during the receipt of stimulation. There was a 2 minute 30 second rest interval between each saliva collection. The post-stimulation saliva was collected after 5 minutes from the end of the stimulation (Fig. 1c). Saliva collections were performed seven times: before stimulation (S1), during stimulation (S2-S6), and 5 minutes after the end of stimulation (S7). As indexes of vital reactions, physical items including changes in systolic blood pressure, diastolic blood pressure, and heart rate were measured as indexes of the cardiovascular system in this study. After stimulation, subjects were instructed to record VAS for evaluating discomfort or pain.

-Cortisol

Changes in cortisol levels were measured using YK241 Cortisol (Saliva) EIA kit (Yanaihara Research Institute, Shizuoka, Japan) as a physiological correlate of stress activation. The quantity of cortisol was indicated as the difference between S1 and S2-7. Each difference was assigned to C1-6. For example, the difference between S1 and S4 was assigned to C3.

-Statistical analysis

Statistical analysis was performed using SPSS ver. 20.0 (SPSS, Chicago, IL, USA). Paired t-test was used for comparisons between the pre- and post-stimulation body temperature and blood pressure. Scheffe’s multiple comparison was used for comparison among three stimulation conditions. Where collected data were normally distributed, LSD multiple comparison test was applied. In the case of non-normal distribution of collected data, the Friedman test was applied. Significance level was set at *p*<0.05.

## Results

-Subjects and stimulation conditions

The characteristics of subjects and the stimulation conditions are shown in Table 1. Body temperature, systolic blood pressure, and diastolic blood pressure are shown in Table 2. The mean body temperature did not change between before and after stimulation in all conditions. The systolic blood pressure decreased after stimulation in all stimulation conditions. However, no significant difference was noted among all stimulation conditions. The diastolic blood pressure increased after stimulation in all stimulation conditions. However, no significant difference was noted among all stimulation conditions.

**Table 1:**
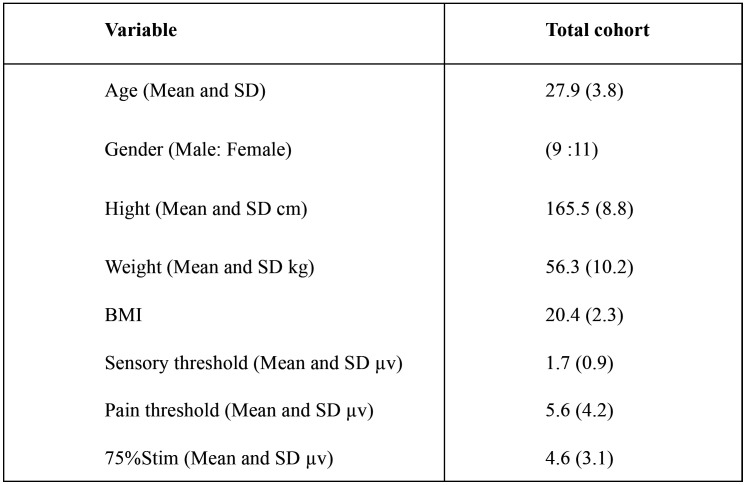
Characteristics of subjects and intensity of stimulation.

**Table 2:**
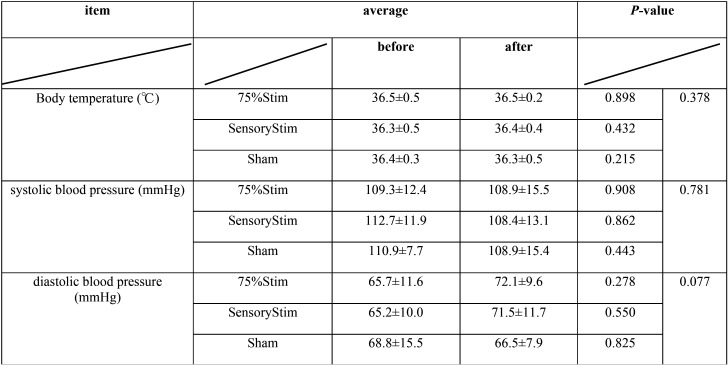
Change of body temperature and blood pressure.

-Salivary flow

**Figure 2:**
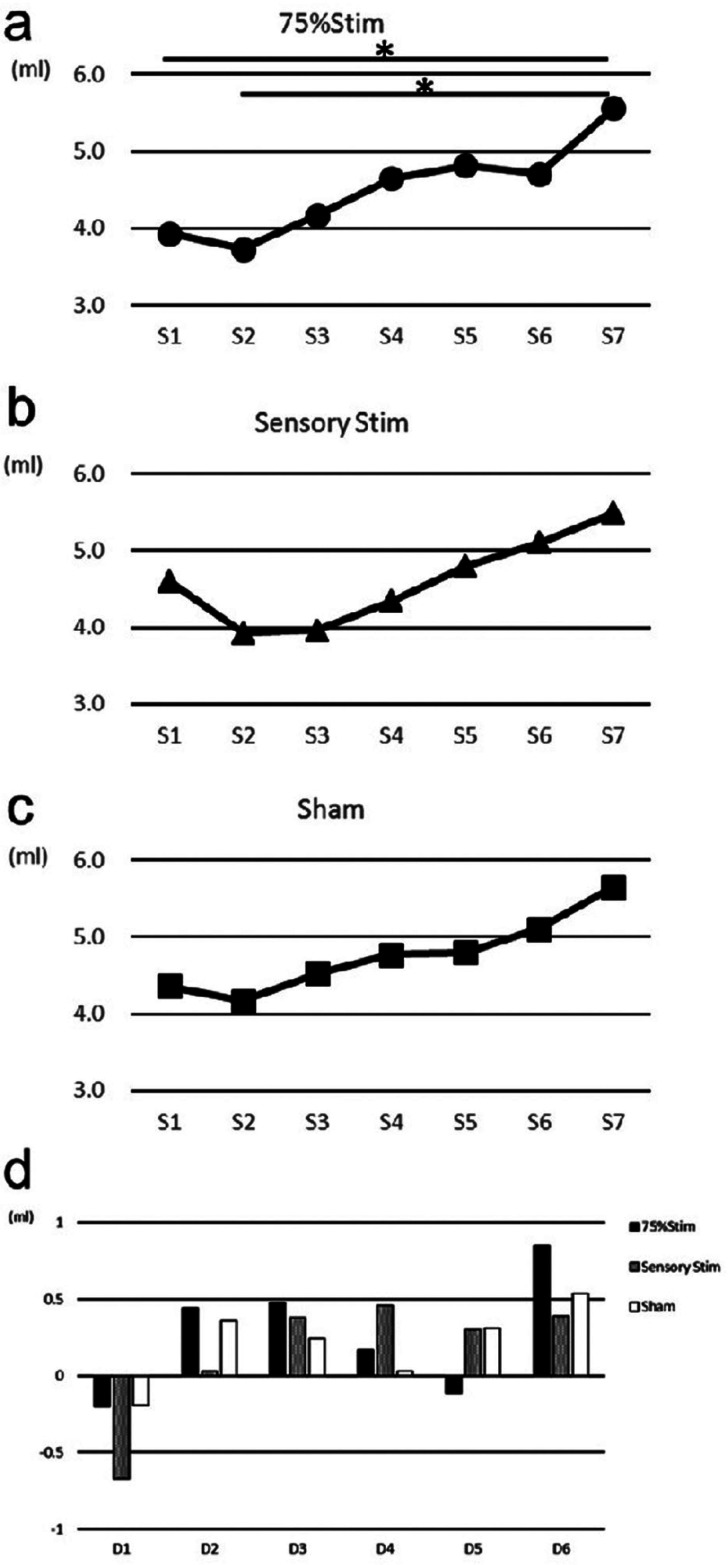
a-c) Changes in quantity of saliva flow. The quantities of saliva flow for S1-S7 were compared among each stimulation conditions. a) 75% Stim, b) SensoryStim, c) Sham. There was a significant difference between S1 and S7, as well as S2 and S7 in 75% Stim. d) Changes in quantities of saliva. There were no significant differences among all conditions.

Salivary flow before, during, and after stimulation for each stimulation condition are shown in Figure 2a-c. Significant differences were noted between S1 and S7, as well as between S2 and S7 in 75% Stim. Further, a tendency that the salivary flow with S3 was decreased compared with S7 in 75%Stim was noted. A tendency for salivary flow with S2 and S3 were decreased compared with S7 in SensoryStim was noted.

The changes in salivary flow among consecutive stimulations by each stimulation were labeled: S2-S1(D1), S3-S2(D2), S4-S3(D3), S5-S4 (D4), S6-S5(D5), and S7-S6(D6)(Fig. 2d). No significant difference was observed among all conditions. A significant difference was noted between D1 and D4 in SensoryStim.

-Pulse rate

**Figure 3:**
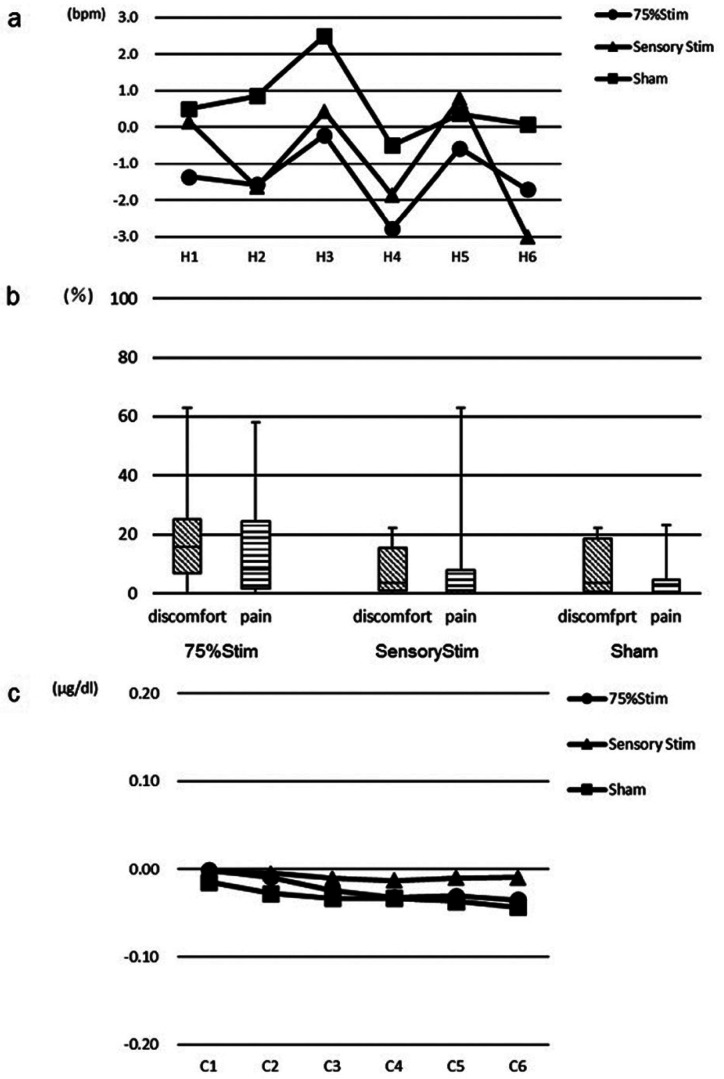
a) Changes in pulse rate. There were no significant differences among all conditions. b) Results of VAS score for discomfort and pain. The median VAS score for discomfort and pain showed a low value for all conditions. c) Changes in quantity of cortisol. No significant changes were noted among all conditions.

The pulse rate recorded every 10 minutes from the start of stimulation to the end of the session are shown in Figure 3a. The changes in pulse rate of the start of stimulation by each stimulation were labeled: H1-H6. No significant differences were noted among all conditions.

-Discomfort and pain 

The median VAS scores are shown in Figure 3b. With regards to VAS score, subjective evaluation revealed a low value for conditions of Sensory Stim and Sham for both discomfort and pain. A similar tendency was noted for the condition of 75% Stim.

-Cortisol

The quantities of cortisol are shown in Figure 3c. No significant differences were noted among all conditions.

## Discussion

This study investigated the effects of NMES with low frequency stimulation on saliva flow, salivary component, and saliva quality when applied to the suprahyoid muscles. This study used three different strengths of stimulation including Sham, Sensory Stim, and 75% Stim. With regards to saliva flow, significant differences were noted between S1 and S7, as well as between S2 and S7 only in 75% Stim. These results suggested that the increase in saliva flow may be promoted by stronger stimulation during NMES. In a previous study, Hersheal *et al.* (26) reported that saliva flow quantity increased following transcutaneous electrical nerve stimulation (TENS) with low frequency applied to the parotid gland in healthy adults. As a similar type of stimulation was applied in that study, their results were similar to ours. Further, in their report, the targeted area was the parotid gland. The authors proposed that neuronal mechanisms promoting salivation were stimulated directly by low frequency stimulation. The targeted area in our study was the submandibular gland area. As such, the stimulation areas were different. However, neuronal mechanisms promoting salivation in the submandibular gland may be stimulated directly by low frequency stimulation, similar to their report.

Hasegawa *et al.* (27) reported that salivation was promoted by application of interferential current stimulation (IFCS) to the submandibular and sublingual gland regions in patients with dry mouth. IFCS uses an electric current with two different frequencies of middle-frequency range over 2,000 Hz. Their study used 2,000 Hz and 2,050 Hz currents to generate an amplitude modulation of 50 Hz. IFCS was performed using two pairs of electrodes that were applied directly to a patient’s body. Based on their results, we hypothesized that salivation may be promoted by low-frequency stimulation. The results of our study suggested that the increase in saliva flow may be promoted by stronger stimulation after NMES. Both NMES in our study and IFCS were applied to the submandibular gland region, albeit at different amplitudes. The amplitude of NMES was low frequency stimulation. Conversely, the amplitude of IFCS was medium frequency stimulation. High amplitude reaches deeper regions than does low amplitude stimulation (28). However, the submandibular gland is located superficially. Therefore, NMES stimulation reached the submandibular gland, and saliva flow may have been changed by the low-frequency stimulation in our study.

Blood pressure and salivary cortisol levels were consistent across the three stimulation conditions. No significant difference was noted in any two stimulation conditions. The quantities of cortisol in saliva were also very consistent through C1 to C6, and no significant difference was noted in any two periods. Kim *et al.* (29) reported that blood pressure decreased when stress was reduced. Both systolic and diastolic blood pressures are related to stress, as both blood pressures decreased concurrently when stress is reduced. There were no significant differences between the reductions in both systolic and diastolic blood pressure in this study. In addition, there were no significant differences in cortisol quantity among three stimulation strengths and between any two stimulation strengths used in this study. Subjective evaluation indicated low values under Sensory Stim and Sham for both discomfort and pain. In addition, relatively low values for both discomfort and pain were observed under 75% Stim. These results suggest that the stress induced in subjects was small when the standard stimulation strength of NMES was applied.

The results of this study suggested that an increase in saliva flow may be promoted by stronger stimulation after NMES. Moreover, the increase in saliva flow may promote continued salivation after NMES. However, in this study, the saliva after NMES was collected only after 5 minutes from the end of NMES. It remains unclear to what timepoint salivation after NMES continues. Future studies should consider temporal changes in salivation.

In this study, the increase in saliva flow was noted similar to that in a previous study in which medium frequency stimulation was applied to the submandibular and sublingual gland regions. Compared to the submandibular gland, the parotid gland is present in more superficial layers (30). In this study, we did not evaluate subjects’ fat thickness of the submandibular region and salivary gland location that may have affected the depth of electrical stimulation, in turn altering salivary flow; this is an issue that future studies should consider.

In this study, NMES for the submental region affected the amount of saliva flow. An increase in saliva flow was promoted after NMES. The results of this study suggested that NMES may be performed with minimal stress to patients. Therefore, NMES has the potential to be conducted with low stress and indirect training in dysphagic patients. Further, NMES may have significant effects on patients with xerostomia.
